# Plastic Optical Fiber Displacement Sensor Based on Dual Cycling Bending

**DOI:** 10.3390/s101110198

**Published:** 2010-11-15

**Authors:** Jao-Hwa Kuang, Pao-Chuan Chen, Yung-Chuan Chen

**Affiliations:** 1 Department of Mechanical and Electro-Mechanical Engineering, National Sun Yat-Sen University, Kaohsiung, 80424, Taiwan; E-Mail: kuang@mail.nsysu.edu.tw (J.H.K.); d933020009@student.nsysu.edu.tw (P.C.C.); 2 Department of Vehicle Engineering, National Pingtung University of Science and Technology, Pingtung, 91201, Taiwan

**Keywords:** plastic optical fiber, cycling bending, displacement fiber sensor

## Abstract

In this study, a high sensitivity and easy fabricated plastic optical fiber (POF) displacement sensor is proposed. A POF specimen subjected to dual cyclic bending is used to improve the sensitivity of the POF displacement sensor. The effects of interval between rollers, relative displacement and number of rollers on the sensitivity of the displacement sensor are analyzed both experimentally and numerically. A good agreement between the experimental measurements and numerical calculations is obtained. The results show that the interval between rollers affects sensitivity most significantly than the other design parameters. Based on the experimental data, a linear equation is derived to estimate the relationship between the power loss and the relative displacement. The difference between the estimated results and the experimental results is found to be less than 8%. The results also show that the proposed POF displacement sensor based on dual cyclic bending can be used to detect displacement accurately.

## 1. Introduction

Optical fibers are widely used in communication systems because of their low attenuation, light weight, higher data transmission rates and no electromagnetic influence [1]. In recent years, different types of optical fiber sensors for measuring displacement, temperature, pressure and other possible applications have been developed [2–4]. Compared with glass optical fibers (GOFs), plastic optical fibers (POFs) have higher numerical aperture, easier connectivity, are cheaper and display more flexibility [4–8]. Hence, POFs are more suitable for use in sensing devices. Donlagic [9] indicated that optical fiber sensors can usually be divided into extrinsic fiber optic sensors [10,11] and intrinsic fiber optic sensors [12–15]. Extrinsic fiber optic sensors are not useful for many purposes because the intensity of the light is changed by contaminated mirrored surfaces [14]. Intrinsic fiber optic sensors have higher signal stability because the output power can be changed according to the variation of measured parameter. The fabrication of optical fiber sensors based on macro- or micro-bending is simple, cheap and provides high signal stability. Furthermore, they can provide a wider measurement range and higher sensitivity [15].

In theoretical and practical applications, the prediction of radiation loss in bent optical waveguides is an important topic [16]. Different purposes for bent optical fiber sensors have been proposed by many researchers [16–22], such as axial strain and curvature measurements [16–18], crack detection and vertical deflection monitoring [19], liquid-level measurement [20], signal compensation [21], humidity monitoring [22], etc. In order to improve the bend sensitivity of the optical fiber sensors, a segment of the POF cross-sectional profile was removed or increased the structural imperfections [14,16,17,19,20]. However, a POF sensor with structural imperfections may cause stress concentration or structural defects after a long time in service and this results in signal monitoring errors, especially for the POF with tooth shape imperfections. It is known that sensitivity improvment is not obvious for a POF with a bent radius. Thus, how to increase the sensitivity of the sensor with no imperfections in the POF is an important issue. Currently, few publications have investigated the sensitivity of sensors based on cyclic bending [9,23–30]. For example, Yin *et al*. [23] presented the application of a fiber specklegram sensor (FSS) to fine angular alignment. The results indicated that FSS is highly sensitive to fiber perturbations, due to which the sensitivity measurement increases as the NA of the sensing fiber increases. Kulkarni *et al*. [24] investigated the use of a novel POF sensor for measuring force in terms of weights. It was shown that increasing the corrugation pitch of the deforming plates enhanced microbend sensitivity. Sanz *et al*. [25] developed a POF force sensor by placing the POF in a snake-shaped mould. The results indicated that in the absence of force the POF remained undisturbed whereas with an external pressure the fiber inside the mould was stressed. Losada *et al*. [26–28] showed that the application of strain to multiple curvatures POFs caused a reduction in the bandwidth and an increased power loss. Jiménez *et al*. [29] explored a plastic optical fiber-based displacement sensor. The sensor was fabricated using a POF wrapped around a flexible cylinder. The results showed that the sensitivity can be improved by predeforming the cylinder or by decreasing its radius. Lomer *et al*. [30] presented a quasi-distributed level sensor based on an extra attenuation arisen from bending and polishing plastic optical fibers. The level sensor takes advantage of the imperfection and the different revolving spindle to increase the sensitivity.

In this study, a highly sensitive plastic optical fiber displacement sensor (POF displacement sensor) based on cyclic bending is analyzed both experimentally and numerically. The POF sensor is pressed by cylindrical models without surface damage. Dual bending model is used to increase the sensitivity of the POF displacement sensor. The effects of number of rollers, the distance between top and bottom plates, and the interval between two rollers on sensitivity of the sensor are discussed.

## 2. POF Displacement Sensor Design

**Figure 1.**
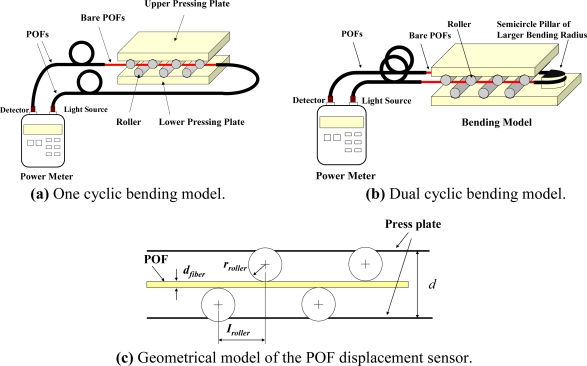
Experimental setup used to measure power loss in cyclic bending POF sensor.

In this study, a highly sensitive POF displacement sensor based on cyclic bending is proposed. Figure 1 shows the experimental setup used to measure the power losses of a cyclic bent POF sensor. As shown, the arrangement includes POFs, a cycling bending model, and an optical power meter (Photom, model 205A) with a detector and a light source. In the experiments, the illumination source used is a light-emitting diode (LED) with a central wavelength of 660 nm. The launch NA of the LED is 0.5. As we know, when light goes through a cyclic bent POF, the power loss occurs. The POF specimen used is a step index type SH-4001 fiber (Mitsubishi Rayon Company Ltd.) with a coating diameter of 2.2 mm, a cladding diameter of 1 mm, a core diameter of 0.98 mm, and a numerical aperture (NA) of 0.5. The refractive index of core and cladding are 1.492 and 1.402, respectively. The core, cladding and coating of these POFs are fabricated from polymethyl methacrylate (PMMA), fluorinated polymer and low-density polyethylene (LDPE), respectively. Figure 1(a,b) shows the configuration of a POF specimen subjected to one cyclic bending and dual cyclic bending, respectively. In the following, they are named as one cyclic bending POF sensor and dual cyclic bending POF sensor, respectively. The dual cyclic bending model is used to improve the sensitivity of the sensor. The first cyclic bending route is from the light source to the one-half cylinder. The second one is from the one-half cylinder to the light detector.

The geometrical model of the POF displacement sensor is shown in Figure 1(c). The parameters studied include the number of rollers (*R_N_*), the interval between two rollers (*I_roller_*) and the distance between the two pressing plate (*d*). As shown in Figure 1(c), the smallest interval in the bending model is limited to 9 mm. If the smallest interval is used in this model, the fiber will sustain a larger plastic deformation and this may result in damage to the core surface. In addition, a larger interval will result in a smaller power loss. In order to investigate the effect of interval on the power loss and avoid smaller power loss, three intervals, *i.e.*, 12 mm, 14.5 mm and 18 mm are discussed in this study. In order to investigate the sensitivity of the POF sensor, different numbers of rollers, from four up to seven, are employed in this study. Although, the diameter of the rollers is an important parameter, a smaller roller radius can cause a plastic deformation and result in residual stress stored in the POF sensor. Thus, a roller with a radius of 4 mm is considered in this study. The rollers are fixed by a V-ditch. The initial distance *d_0_* between the two pressing plates before bending is 13.3 mm. The plate distance *d* is varied during bending deformation process. A relative displacement Δ*d* is defined as *d*_0_ − *d*. The parallelism between the upper pressing plate and the lower pressing plate also affects the relative displacement. Hence, in these experiments, the upper pressing plate is restricted to move only up and down by four smaller cylinders in four corners of the developed POF sensor to control the parallelism. More than three experimental tests are performed to explore the effect of each parameter on the POF displacement sensor.

## 3. Results and Discussion

**Figure 2.**
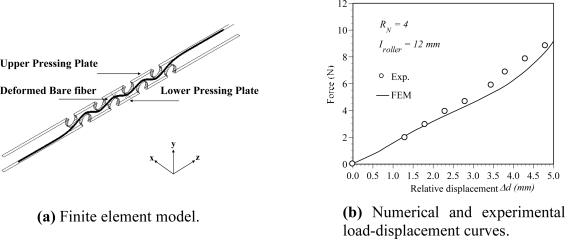
Three-dimensional finite element model of a cyclic bent POF specimen and load-displacement curve.

**Figure 3.**
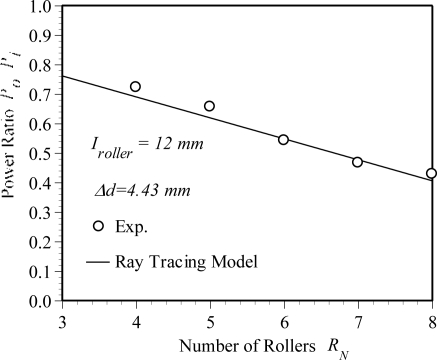
Variation of the power ratio with number of rollers.

Both the experimental measurement and the numerical analysis are used to explore the sensitivity of the POF displacement sensor. In this study, the bending shape of pressed POF sensor is obtained from an elastic-plastic three-dimensional finite element simulation. The simulations are performed using a commercial finite element package (ABAQUS). The power losses then can be calculated by substituting the deformed POF geometrical dimensions into the optical numerical software-ZEMAX. In the numerical simulation, a 3-D ray tracing model is used. The light input is assumed to be uniformly distributed over the core cross section because the light source used in experiment is a LED. The ray tracing model includes meridional and skew rays. The maximum ray's angle with respect to the fiber center axle is 20°. The power of the POF sensor before and after deformation are denoted as *P_i_* and *P_0_*, respectively. Various numbers of rays are selected for the convergence test of the power loss calculation. The power ratio *P_i_*/*P_0_* calculated from the case with ray numbers of 100,000, 200,000 and 1,000,000 are 0.702, 0.701 and 0.703, respectively. In order to save the computation time, the number of rays is taken as 100,000 in this study. Figure 2(a) shows the finite element meshes of the bent POF sensor. Due to the symmetry of the POF geometry, only one half of the model is considered in the finite element analysis. In performing the analysis, an assumption is made that the pressing plate is a rigid body. In general, the finite element model involves approximately 28,800 eight-node brick elements and 37,467 nodes. The mechanical properties used in finite element simulations are listed in Table 1. Figure 2(b) presents the load-displacement relationship obtained from the experiments and numerical simulations. In this figure, the symbols represent the experimental results and the solid line is the numerical results obtained from the proposed elastic-plastic finite element model. In this case, the number of rollers *R_N_* and the interval *I_roller_* are taken as 4 and 12 mm, respectively. It is clear that a good agreement exists between the two sets of results. The uncertainty is less than 5% of their nominal values. Figure 3 shows the variation of the power ratio *P*_0_*/P_i_* with the number of rollers. In Figure 3, the pressing plate with a relative displacement Δ*d* of 4.43 mm is used. It is noted that one cyclic bending model is considered for power ratio calculation. In this figure, the solid line and symbols represent the numerical results obtained from the ray tracing model and experimental measurements, respectively. The results indicate that a good agreement is obtained between the two sets of results. From the results shown above, it can be concluded that the proposed finite element and ray tracing combined model can predict the power loss of the deformed POF sensor accurately. The results also show that the power ratio is decreased linearly with the increase of number of rollers.

**Figure 4.**
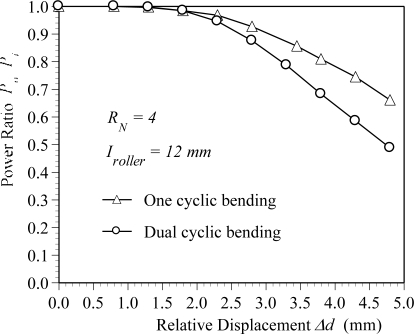
Variation of the power ratio *P_0_*/*P_i_* with the relative displacement.

**Figure 5.**
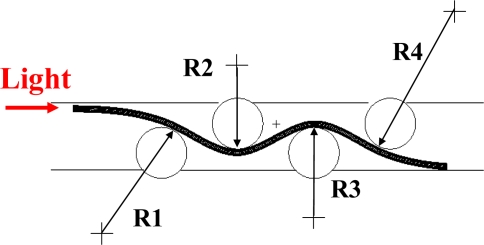
Deformed POF displacement sensor.

**Figure 6.**
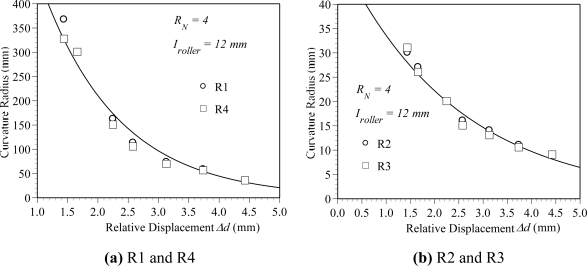
Variation of the curvature radius with the relative displacement for *R_N_* = 4 and *I_roller_* = 12 mm.

Figure 4 shows the variation of the power ratio *P_0_*/*P_i_* with the relative displacement for *R_N_* = 4 and *I_roller_* = 12 mm. It can be seen that no power loss is observed when the relative displacement is less than 1.5 mm. This means that the bend deformation of the POF sensor in this range of relative displacement cannot affect the transmission power. The result also indicates that the power loss increases with the relative displacement when the relative displacement is larger than 1.5 mm. The result can be explained by figures (5) ∼ (9). Figure 5 shows the deformed POF displacement sensor. It can be observed that there are four different curvature radii, *i.e.*, R1∼R4, on the deformed POF sensor. As shown, the curvature radius R1 is close to the input side of the light source, and R4 is close to the output side of the light source. Various element meshes are performed for the convergence test of the curvature radius R2, as shown in Figure 5. The mesh ratio is defined as the ratio of the element length *l_e_* along the fiber to the core diameter *d_co_*. The mesh ratios used are 2.869, 1.435, 0.729 and 0.364. The relative displacement Δ*d*, interval *I_roller_* and roller number *R_N_* are 4 mm, 12 mm and 4, respectively, in these cases. The curvature radii obtained from the mesh ratios 2.869, 1.435, 0.729 and 0.364 are 12.64, 10.52, 10.0 and 9.77 mm, respectively. The results indicate that the variation in curvature radius R2 is not obvious for the mesh ratio greater than 0.729. Compared with the results obtained from the mesh ratio of 0.729 and 0.364, the difference is less than 3%. In order to save the computation time, the mesh ratio of 0.729 is used in this paper. Figure 6 shows the variation of the curvature radius obtained from the finite element simulations with the relative displacement for one cyclic bending model. It is observed that the curvature radii R1∼R4 decrease with an increasing relative displacement. The result also indicates that for a constant relative displacement, the value of R1 is close to R4 and R2 is close to R3. Both the curvature radii R1 and R4 are much larger than R2 and R3. For example, the curvature radii R1 and R2 are 35 mm and 9 mm, respectively, as the relative displacement Δ*d* is 4.5 mm. The literature [34] indicated that the power loss depended on the curvature radius of the specimen. The smaller the curvature radius is, the larger the power loss is. In addition, for a dual cyclic model, the curvature radii of the deformed POF sensor on first rollers and the second rollers are the same due to the geometrical symmetry, as shown in Figure 1. Because the light power distribution is changed by the first rollers, this will lead to more light power loss in the second rollers.

**Figure 7.**
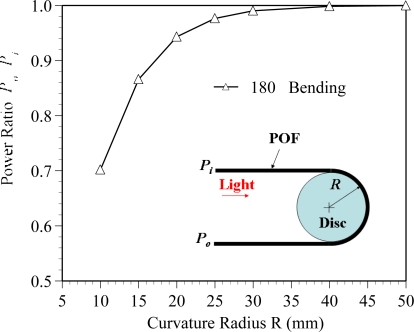
Variation of the power ratio *P_0_/P_i_* with the curvature radius for POF specimen subjected to 180 degree bending

Figure 7 plots the variation of the power ratio with the curvature radius for the POF specimen with 180 degree bending. The results are obtained from an experimental measurement. It is seen that for R > 30 mm, the power ratio is equal to one. It means that the power loss is not obvious when the curvature radius is larger than 30 mm. The power loss increases obviously with reduced curvature radius as *R* is less than 30 mm. As shown in Figure 6(a), the curvature radius of R1 or R4 is remained larger than 30 mm for the relative displacement is less than 4.5 mm. The results confirm that the power loss is nearly independent of R1 and R4 as the relative displacement is increased from 0 to 4.5 mm.

**Figure 8.**
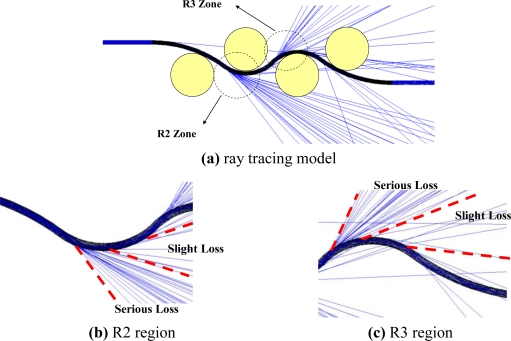
Ray path of ray tracing model in deformed POF sensor.

**Figure 9.**
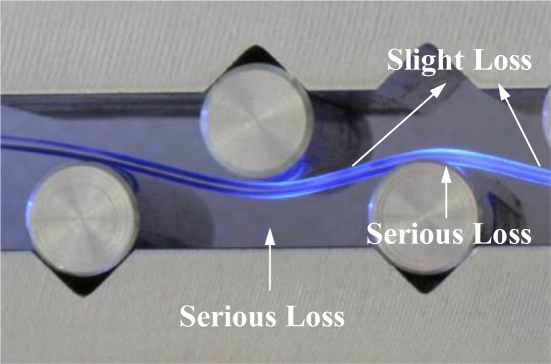
Light loss in a deformed POF sensor.

Thus, the power loss of the proposed POF displacement sensor is mainly attributed to R2 and R3, because the curvature radii R2 and R3 are less than 30 mm as the relative displacement is increased from 1.5 to 4.5 mm, as shown in Figure 6(b). The ray path of the ray tracing model for the POF sensor subjected to cyclic bending is shown in Figure 8. In this figure the ray path in a 3-D ray tracing model with a relative displacement Δ*d* = 4.5 mm is presented. In order to show the ray paths in the deformed POF sensor clearly, only 1,000 rays are used in this figure. When rays transmit from the straight region or region with a large radius of curvature (R > 30 mm) to the cyclic bending region, it can be seen that most of the optical power is lost at the bending regions with curvature radii R2 and R3. The picture shown in Figure 9 also indicates the behavior of power losses in bending POF sensor. It can be concluded from the results shown in figures 5 ∼ 9 that no power loss is observed when the relative displacement is less than 1.5 mm, as shown in Figure 4. Meanwhile, the results confirm that when the relative displacement is larger than 1.5 mm, the power loss increases with the relative displacement. In addition, compared with one cyclic bending POF sensor shown in Figure 4, it is obvious that the power loss in dual cyclic bending POF sensor increases, *i.e.*, sensitivity increase. For instance, given a relative displacement Δ*d* = 4.3 mm, the power ratios obtained from one and dual cyclic bending POF sensor are 0.74 and 0.58, respectively. The power ratio is reduced by 0.16 (*i.e.*, 0.74–0.58). It can be concluded that the dual cyclic bending POF displacement sensor can have a higher sensitivity than the one based on one cyclic bending.

**Figure 10.**
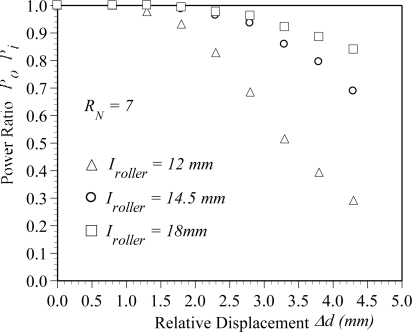
Variation of the power ratio *P_0_*/*P_i_* with respect to the relative displacement for various intervals.

Figure 10 plots the variation of the power ratio with respect to the relative displacement for various intervals. In this figure, a dual cyclic bending POF sensor with seven rollers is used to measure the power losses. Three different intervals *I_roller_*, *i.e.*, 12 mm, 14.5 mm, and 18 mm, are explored. In Figure 10, the symbols represent the experimental results. The results show that, for a constant relative displacement, the power loss reduces with increasing interval. For example, given a relative displacement of Δ*d* = 4.3 mm, the power ratios obtained from interval *I_roller_* = 12 mm and 18 mm are 0.29 and 0.84, respectively. The power ratio increases by 0.55 (*i.e.*, 0.84–0.29). It can be attributed to that the reduction of interval will cause the bend radius of the POF sensor close to the roller radius and result in large power loss. Due to a smaller interval results in a higher sensitivity of the POF displacement sensor, an interval *I_roller_* = 12 mm is used to explore the effect of number of roller on the power ratio in the following.

**Figure 11.**
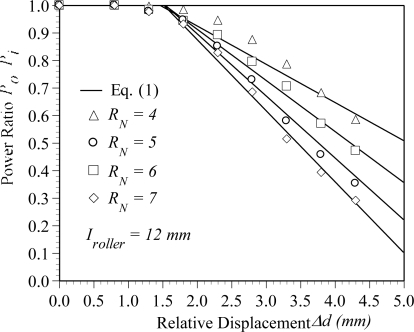
Variation of power ratio *P_0_*/*P_i_* with relative displacement as a function of number of roller for a dual cyclic bending POF sensor.

Figure 11 shows the variation of the power ratio with the relative displacement as a function of the number of rollers for a dual cyclic bending POF sensor. The symbols represent the experimental results.

Once again, the results confirm that no power loss is observed when the relative displacement is less than 1.5 mm, even more rollers are used. Meanwhile, it can be seen that when the relative displacement is larger than 1.5 mm, the power loss presents a nearly linear dependence with the relative displacement. The results also show that the power loss increases with an increasing number of rollers. More rollers imply that the POF sensor undergoes more geometric deformations, and thus the radiation loss increases with the number of rollers. For example, as the relative displacement is increased from Δ*d* = 1.8 mm to Δ*d* = 4.3 mm, the power ratio reduces by 0.4 (*i.e.*, 0.98–0.58) for the case of *R_N_* = 4. However, the power ratio reduces by 0.64 (*i.e.*, 0.93–0.29) for the case of *R_N_* = 7. The results show that the sensitivity of the POF displacement sensor can be improved by adding rollers. Applying a linear curve fitting procedure to the data shown in Figure 11, the power ratio *P_0_*/*P_i_* can be related to the relative displacement Δ*d* by the following expressions:

(1a) $$P_{o}/P_{i}=1,\;\;\;\mathrm{as}\;\;\Delta d\leq 1.5\mathrm{mm},\;\text{for}\;I_{\mathrm{roller}}=12\;\text{mm}$$

(1b) $$P_{o}/P_{i}=A\left(\Delta d-1.5\right)+1,\;\;\;\;\mathrm{as}\;\;\Delta d>1.5\mathrm{mm},\;I_{\mathrm{roller}}=12\;\text{mm}$$

where *A* is a function of rollers and can be expressed as:

(1c) $$A=-3.74*10^{-2}\left(R_{N}\right)+1.8*10^{-3}$$

Note that the unit of Δ*d* in Equation (1) is *mm*. The uncertainty of parameter *A* is less than 4.4% of their nominal values. Equation (1) can be used to predict the power loss with the information of relative displacement. The lines shown in Figure 11 represent the results obtained from Equation (1). Compared to the experimental results shown in Figure 11, the maximum difference between the experimental data and the results estimated by Equation (1) is less than 8%. The chi square values calculated for the cases of *R_N_* = 4, *R_N_* = 5, *R_N_* = 6, and *R_N_* = 7 are 1.56 × 10^−2^, 6.36 × 10^−3^, 6.43 × 10^−3^ and 5.20 × 10^−3^, respectively, and all the corresponding degrees of freedom are 7. Therefore, it can be concluded that the proposed Equation (1) can predict the power loss of the POF displacement sensor accurately.

Compared with the results shown in figures 10 and 11, the effect of the interval on the sensitivity of the POF sensor is higher than the number of rollers.

## 4. Conclusions

This study has conducted experimental and numerical investigations into the effects of relative displacement, number of rollers, and interval between two rollers on the sensitivity of a cycling bending POF displacement sensor. The experimental measurements and numerical results indicate that the proposed POF sensing model is feasible for measuring the displacement. The results show that the POF displacement sensor based on cycling bending is significantly affected by the number of rollers, relative displacement and the interval. The power ratio reduces significantly as the number of roller is increased and the interval is decreased. The results of a basic ray tracing analysis show that most of the optical power is lost at the first turn and its turning points. Based upon the experimental results, an empirical expression is formulated to relate the power loss and the relative displacement. The maximum deviation between the predicted power loss obtained from the proposed equation and the experimental result is found to be less than 8%. Thus, the suitability of the relative displacement as a means of predicting the power loss in deformed POF sensors is confirmed. Overall, the presented results confirm the viability of POF sensors based on cyclic bending and suggest that the sensitivity of such devices can be enhanced by increasing the number of rollers or decreasing the interval between rollers. The potential applications of the developed cyclic bending-POF sensing element can be found in measuring small displacements at certain areas or alarming landslide for its high sensitivity under deformation.

## Figures and Tables

**Table 1.** The mechanical properties of the POF specimen used in finite element model.

**Properties**	**PMMA [31]**	**PTFE [32]**	**LDPE [33]**

*E* (MPa)	3000	215	200
σ*_v_* (MPa)	56	11.3	9.8
σ*_ult_* (MPa)	68	55	17.15
*ν*	0.4	0.46	0.49
